# Simultaneous molecular detection of *Mycobacterium tuberculosis* and multidrug resistance using CRISPR-AaCas12b-based nucleic acid assay

**DOI:** 10.3389/fcimb.2026.1844184

**Published:** 2026-05-19

**Authors:** Yujiao Hu, Dan Zhao, Yanjun Diao, Congxia Bai, Ke Zhou, Fang Huang, Rui Li, Xiaoyan Hao, Hao liu, Jiayun Liu, Lei Zhou

**Affiliations:** 1 Department of Clinical Laboratory Medicine, Xijing Hospital, Fourth Military Medical University, Xi’an, China; 2 Department of Clinical Laboratory Medicine, Xi’an Chest Hospital, Xi’an, China

**Keywords:** tuberculosis (TB), multidrug-resistant tuberculosis (MDR-TB), CRISPR-AaCas12b, lateral flow chromatography (LFC), *rpoB* 1349C>T, *katG* 944G>C

## Abstract

**Objectives:**

To address the unmet need for rapid, accurate diagnosis of *Mycobacterium tuberculosis* (MTB) and multidrug-resistant tuberculosis (MDR-TB), we developed and validated a clustered regularly interspaced short palindromic repeats-associated protein (CRISPR-Cas)-based diagnostic assay.

**Methods:**

A multiplex-recombinase polymerase amplification (RPA) coupled CRISPR-*Alicyclobacillus acidiphilus* Cas12b (AaCas12b) assay was established for simultaneous detection of MTB by targeting the specific insertion sequence *IS6110* and the two most common drug resistance mutations, *rpoB* 1349C>T for rifampicin resistance and *katG* 944G>C for isoniazid resistance. The assay supported dual-readout signal detection using both a fluorescent platform and lateral flow chromatography (LFC). Its diagnostic performance was evaluated in 48 clinical samples using WHO-recommended GeneXpert MTB/RIF, phenotypic drug susceptibility testing (pDST), and sequencing as reference standards.

**Results:**

The multiplex-RPA CRISPR-AaCas12b assay showed a limit of detection (LoD) of 1.5 CFU/mL for MTB detection, with a sensitivity of 97.1% and a specificity of 100% using culture as the reference standard, and a total turnaround time of 30 min (20 min for RPA and 10 min for CRISPR cleavage). For MDR-TB-related mutations, the assay achieved a sensitivity of 94.1% and a specificity of 100% for *rpoB* 1349C>T, and 94.7% sensitivity and 93.1% specificity for *katG* 944G>C, using sequencing as the reference standard. Notably, the LFC-integrated assay maintained comparable diagnostic accuracy with a total turnaround time of 35 min (20 min for RPA, 5 min for CRISPR cleavage, and 10 min for lateral flow strip reading).

**Conclusion:**

The established multiplex-RPA CRISPR-AaCas12b assay enables simple, accurate, and sensitive detection of MTB and common mutations associated with MDR-TB. With a rapid, simplified workflow and low resource requirements, this approach holds considerable potential for point-of-care testing in resource-limited settings, thus facilitating improved surveillance and control of TB and drug-resistant TB.

## 1 Introduction

Timely and accurate detection of *Mycobacterium tuberculosis* (MTB) and drug−resistant tuberculosis (DR-TB) remains central to effective clinical management, infection control, and global tuberculosis (TB) elimination efforts (Organization GWH,). Conventional mycobacterial culture methods for TB continue to serve as the diagnostic gold standard for active TB, yet its reliance on long incubation periods and limited sensitivity greatly constrains its utility in routine clinical settings. In recent years, the development of nucleic acid-based detection technologies has transformed the landscape of TB diagnosis and DR-TB surveillance. In particular, real-time fluorescence quantitative PCR−based platforms have greatly improved diagnostic speed and performance. Representative systems, including the GeneXpert MTB/RIF (Lessells et al., 2017), Ultra (Dorman et al., 2018; Quan et al., 2024), and XDR (Naidoo and Dookie, 2022) assays, have been widely adopted to enhance the detection sensitivity of TB/DR−TB. Despite these advances, most molecular approaches still require sophisticated equipment and skilled personnel, creating substantial barriers to implementation in low−resource and high−burden settings.

The clustered regularly interspaced short palindromic repeats-associated protein (CRISPR-Cas) system, originally utilized for genome-editing, has emerged as a transformative platform for nucleic acid detection (Myhrvold et al., 2018; Broughton et al., 2020; Patchsung et al., 2020), enabling rapid and accurate detection of a broad spectrum of pathogens without reliance on specialized laboratory infrastructure or sophisticated instruments (Pardee et al., 2016; Nguyen et al., 2022). Building on these advances, CRISPR-Cas systems have recently emerged as promising next−generation tools for rapid and specific TB detection (Jia et al., 2023; Gan et al., 2024). Studies have demonstrated the versatility of CRISPR−based strategies, ranging from CRISPR−Cas12b combined with isothermal amplification for direct detection in sputum (Peng et al., 2023), to integrated PCR−CRISPR platforms for ultra−sensitive analysis of rifampin resistance (Zheng et al., 2024), amplification−free CRISPR−FET sensing systems (Wang et al., 2025), and recombinase-aided amplification (RAA)−coupled Cas13a assays for high−speed MTB identification (Li et al., 2024). Despite the considerable progress that has been made in recent years, the development of CRISPR−based TB diagnostics remains in an early stage. Most current CRISPR-based point-of-care testing (POCT) assays focus on either MTB detection alone or screening for resistance to a single drug, while few assays allow for simultaneously detection of MTB together with both key rifampicin and isoniazid resistance mutations, and many still require specialized detection equipment, limiting their practical utility in resource-limited settings. In addition, most remain at the proof−of−concept stage and require further clinical validation before widespread implementation.

There is therefore a clear, persistent need for a rapid, convenient, and integrated CRISPR-based diagnostic tool capable of identifying both MTB and clinically relevant rifampicin and isoniazid resistance mutations, particularly the *rpoB* 1349C>T for rifampicin resistance and *katG* 944G>C for isoniazid resistance, which are well-established major determinants of drug resistance in clinical practice (Ajbani et al., 2011; Rostamian et al., 2023; Guo et al., 2025). The global distribution of clinically prevalent MTB drug-resistance mutations was summarized in a sunburst plot (**Supplementary Figure 1**), based on curated data from the Drug Resistance Associated Genes database (DRAGdb: Drug Resistance Associated Genes) and The Comprehensive Antibiotic Resistance Database (The Comprehensive Antibiotic Resistance Database). To tackle these challenges, a CRISPR-*Alicyclobacillus acidiphilus* Cas12b (AaCas12b) based diagnostic strategy was developed for the simultaneous detection of MTB and resistance to rifampicin and isoniazid. In this work, soluble AaCas12b protein was prokaryotically expressed and purified, followed by the design and screening of single-guide RNAs (sgRNAs) targeting the MTB-specific insertion sequence *IS6110*, together with the key drug-resistance mutations *rpoB* 1349C>T and *katG* 944G>C. This optimized CRISPR-AaCas12b system was further integrated with a multiplex recombinase polymerase amplification (multiplex-RPA) system, enabling the establishment of a dual-readout detection platform supporting both fluorescent reporting and lateral flow chromatography (LFC). The performance of the established assay was subsequently evaluated using a panel of 48 clinical specimens.

## 2 Materials and methods

### 2.1 Assay workflow

**Figure 1:**
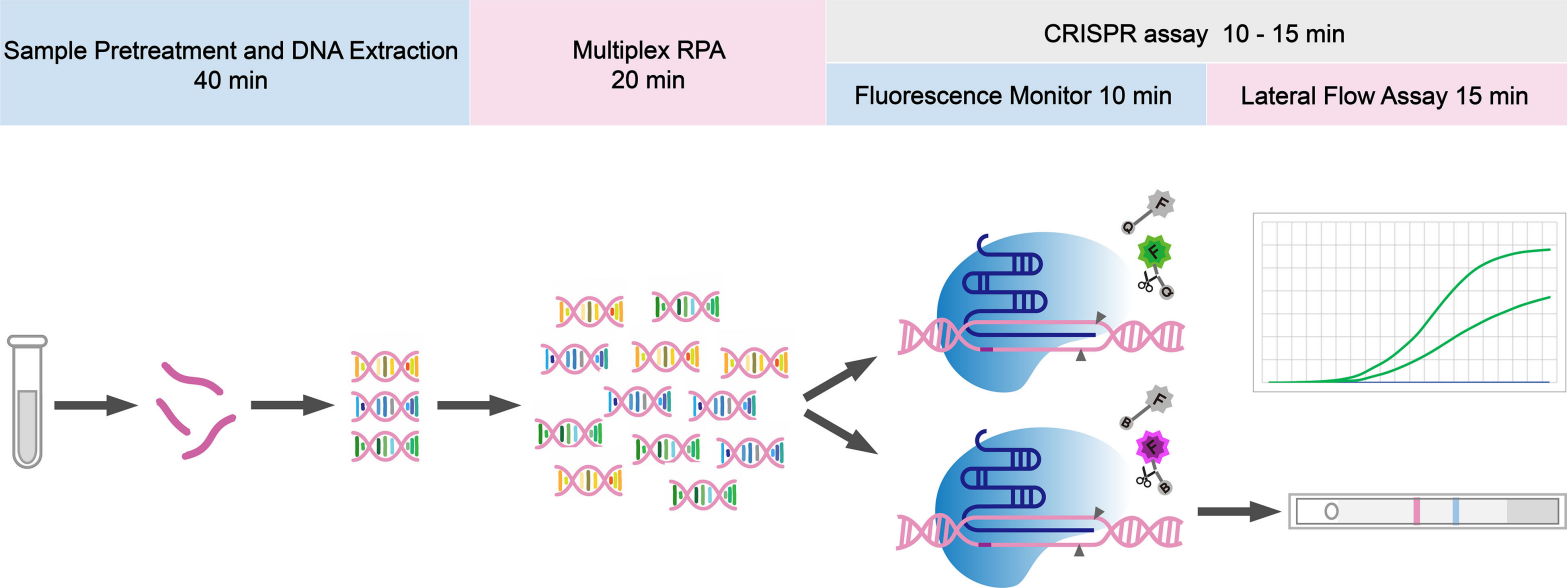
Schematic workflow of the multiplex-RPA CRISPR-AaCas12b assay. The workflow includes sample pre-treatment and nucleic acid preparation (40 min), multiplex-RPA amplification (20 min), and CRISPR-AaCas12b detection with dual readouts (fluorescence: 10 min; lateral flow assay: 15 min).

The overall workflow of the multiplex-RPA CRISPR-AaCas12b assay is shown in **Figure 1**. Briefly, the assay consists of three main steps: (1) sample pre-treatment and nucleic acid preparation, (2) multiplex-RPA amplification, and (3) CRISPR-AaCas12b-based detection using both fluorescence and lateral flow readouts. The total assay time is indicated for each step.

### 2.2 Nucleic acid preparation

RPA primers and single-stranded DNA (ssDNA) reporters containing fluorescence and quenching, or fluorescence and biotin labelled were synthesized by Sangon Biotech (Shanghai, China) (**Supplementary Tables S1**, **S2**).

### 2.3 AaCas12b protein expression and purification

The dsDNA target of AaCas12b (Teng et al., 2018) following a sequence optimized for expression in the form of a pET-30a-derived plasmid was commercially synthesized by GenScript (Nanjing, China). The expression plasmid was then transformed into chemically competent *E. coli* BL21(DE3) cells from TransGen (Beijing, China). Individual colonies were picked and inoculated in OXIOD Luria-Bertani (LB) (Basingstoke, UK) at 37 °C with 200 rpm orbital shaking for 6 h followed by transferring for scale-up until the OD 600 reached 0.6. The culture was then placed on ice for 20 min prior to the addition of 0.25 mM IPTG (Solarbio, Beijing, China) followed by incubation at 16 °C for 16 h. Cells were harvested by centrifugation at 4,000 × g for 10 min, then resuspended in nondenaturing Lysis Buffer from Beyotime Biotechnology (Shanghai, China) containing 1 × PMSF from Beyotime Biotechnology (Shanghai, China). The suspended cells mixture was then broken by ultrasound and centrifuged. The supernatant was filtered through a 0.22 µm Millipore filter (Billerica, MA, USA). Proteins were purified via AKTA Prime Plus Liquid Chromatography System (GE, Sweden) from lysed bacteria using Nickel-Nitrilotriacetic Acid (Ni-NTA) purchased from Beyotime Biotechnology (Shanghai, China) pre-equilibrated with Wash Buffer A (20 mM PB, pH = 8.0) with three concentrations of Imidazole, 5, 10, and 20 mM, respectively, eluted with Elution Buffer B (20 mM PB, 500 mM Imidazole, pH = 8.0). Following ultrafiltration using Millipore ultrafiltration tubes (Billerica, MA, USA), the AaCas12b eluate was dialyzed at 4 °C for 16 h with 50 kDa MWCO Spectrum dialysis kits (Houston, TX, USA).

### 2.4 T7 transcription *in vitro* and sgRNA

The sgRNAs were designed against the sequence following the protospacer adjacent motif (PAM) sequences serving as AaCas12b recognition sites (Teng et al., 2019a). All the sgRNAs used for CRISPR assay were obtained by overnight transcription at 37 °C with T7 RNA Polymerase and purified via magnetic beads purchased from Tolobio (Shanghai, China), whose templates were synthesized by overlap extension PCR (OE PCR). The DNA template for transcription of scaffold sequence of sgRNA was commercially synthesized by GenScript (Nanjing, China). Antisense oligos for transcribing templates were commercially synthesized by Tsingke Biotech (Beijing, China) (**Supplementary Table 3**). The concentrations of sgRNAs were measured using NanoDrop (Thermo Fisher Scientific, Waltham, MA, USA).

### 2.5 RPA assay

The individual RPA assay of *IS6110*, *rpoB* and *katG* genes was performed according to the manufacturer recommended protocol of the multienzyme isothermal rapid amplification basic kit purchased from Amplification (Changzhou, China). The mixtures, in a total 50 µL reaction mix with 2 µL (10 μM) each of forward and reverse primers and 2 µL template DNA, were incubated at 37 °C for 20 min. Agarose gel electrophoresis (AGE) and Sanger sequencing was used to confirm the amplification product of the RPA assay. Then the individual RPA assays were integrated into a multiplex assay, capable of amplifying up to three different targets. Primer sets for *IS6110*, *rpoB* and *katG* genes were optimized in operating conditions of the multiplex-RPA assay. Briefly, the reaction mixtures contained 29.5 µL Buffer A, 1 µL (10 μM) each of forward and reverse primers of *IS6110*, 2 µL (10 μM) each of forward and reverse primers of *rpo*B and *kat*G genes, 2 µL templates, 6 µL ddH_2_O, 2.5 µL Buffer B.

### 2.6 CRISPR-AaCas12b MDR-TB assay

To enable accurate and sensitive detection of single nucleotide mutations associated with rifampicin-resistant tuberculosis (RR-TB) and isoniazid-resistant tuberculosis (Hr-TB), sgRNAs were designed to specifically target the *rpoB* 1349C>T and *katG* 944G>C mutations, while minimizing recognition of the wildtypes. Three complementary strategies (Li et al., 2019; Teng et al., 2019b; Bosch et al., 2021) were used to develop the sgRNAs. First, PAM selection: Leveraging the ability of AaCas12b to recognize both canonical and non-canonical PAMs, which leads to differential target cleavage efficiency. Second, sgRNA length modulation: Varying the length of the sgRNA targeting sequence to adjust the degree of complementarity between the sgRNA and the DNA target, thereby influencing cleavage activity. Third, introduction of deliberate mismatches (IDM): Introducing artificial mismatches into the sgRNA sequence to increase the number of mismatches with wildtype targets relative to mutant targets. Final sgRNAs were selected based on functional validation: those that efficiently cleaved the target mutant sequence but showed no cleavage of the wildtype sequence were chosen for subsequent experiments.

### 2.7 CRISPR-AaCas12b fluorescence assay

Following RPA assay 2 µL of the amplification products were added to the 18 µL CRISPR reaction mix, consisting of 1 × NEBuffer r2.1 (MA, USA), 500 nM of sgRNA, 200 nM of Cas12b, 500 nM of ssDNA reporter and ddH_2_O (**Supplementary Table 2**). This final reaction mixtures were incubated at 37 °C and monitored for fluorescence signal for 10 min collected every 30 s using the Applied Biosystems real-time PCR machine (MA, USA).

### 2.8 Lateral flow chromatography assay

The reaction system and conditions were performed as described in the above CRISPR assays, while 250 nM fluorescent ssDNA reporter biotin labeled was used instead of the quenching reporter for the LFC assay readout (**Supplementary Table 2**). Following a 5-min CRISPR reaction at 37 °C, a sample diluted 10 times using distilled water was loaded onto the test strip purchased from Tiosbio (Beijing, China). Then the test strip was kept at room temperature for 10 min and observed visually.

### 2.9 Strains and clinical specimens

#### 2.9.1 Clinical specimens

To evaluate the Multiplex-RPA CRISPR-AaCas12b system for diagnosing TB and MDR-TB, a total of 48 clinical specimens were included in this study. These specimens were derived from leftover pretreated sputum or bronchoalveolar lavage fluid (BALF) residues from routine clinical TB testing, covering four categories: drug-sensitive TB, DR-TB, non-tuberculous mycobacteria (NTM), and other non-mycobacterial infections (**Supplementary Table 4**). All clinical specimens were fully de-identified to exclude any personal identifying information, protecting the privacy and rights of participants. Specimen processing, including decontamination and digestion, was performed according to the Manual of Clinical Microbiology (Caulfield et al., 2023). Briefly, samples were digested and decontaminated with N-acetylcysteine (NALC)-NaOH solution, neutralized, and concentrated by centrifugation. Crude genomic DNA was prepared by heating the resulting pellet at 100 °C for 10 min to facilitate cell lysis and release nucleic acids for subsequent amplification (Kocagöz et al., 1993). This study was approved by the Medical Ethics Committee of Xijing Hospital of Fourth Military Medical University (Ethics Approval No.: KY20193292) and performed in accordance with the principles of the Declaration of Helsinki.

#### 2.9.2 Control strains

Control strains were obtained from the laboratory stock collection of the Department of Clinical Laboratory Medicine of Xijing Hospital.

### 2.10 Data procession and statistical analysis

ΔRFU was defined as the endpoint fluorescence intensity minus the initial fluorescence intensity of each reaction. For clinical sample validation, the ΔRFU threshold for positivity was determined using the culture-confirmed negative control (NC) samples as the negative reference: threshold = mean ΔRFU of NC + 3×SD of NC. *Mycobacterium tuberculosis* strain H37Rv (*M. tuberculosis* H37Rv) was used as a positive control for MTB identification. For mutation detection, strains confirmed by Sanger sequencing to carry the target mutations, *rpoB* 1349C>T and *katG* 944G>C, served as positive controls. Samples with ΔRFU values above this threshold were considered positive.

Statistical analysis was conducted using IBM SPSS Statistics 25.0. For each sample, data from three technical replicates were averaged, and the mean values were used for statistical comparisons. Student’s t-test was used to compare wildtype and mutant groups within each reaction system. One-way ANOVA followed by Tukey’s *post hoc* test was applied for comparisons among different reaction systems. All tests were two-tailed, and a *p*-value < 0.05 was considered statistically significant. GraphPad Prism 10 was used for data visualization.

## 3 Results

### 3.1 Expression and purification of functional AaCas12b protein

In this study, AaCas12b was collected from the total protein of BL21(DE3) with AKTA Prime Plus Liquid Chromatography protein purification systems by Ni-NTA (**Supplementary Figure 2**). The purified protein solution was stored at −80 °C, and its nuclease activity was verified by the subsequent CRISPR-AaCas12b TB assay.

### 3.2 Multiplex-RPA for simultaneous amplification

Typical scanned images from AGE of individual (targeting the three genes *IS6110*, *rpoB*, and *katG*) and multiplex-RPA of *M. tuberculosis* H37Rv are shown in **Supplementary Figure 3A**. The sensitivity of the multiplex-RPA assay was assessed using different concentrations of serially tenfold diluted *M. tuberculosis* H37Rv. The results shown as visible bands on AGE were observed for the templates diluted to 1.5 CFU/mL, demonstrating a successful multiplex amplification (**Supplementary Figure 3B**).

### 3.3 High sensitivity and specificity of the CRISPR-AaCas12b assay for MTB detection

For accurate detection of MTB, we developed an RPA-coupled CRISPR-AaCas12b assay targeting the *IS6110* insertion element. The *IS6110* region spanning positions 184–203 bp, which contains an NTTG PAM sequence, was selected as the spacer target, based on previous reports demonstrating the high conservation of *IS6110* in the MTB genome (Kechin et al., 2023). The assay achieved a limit of detection (LoD) of 1.5 CFU/mL, as determined using serially diluted *M. tuberculosis* H37Rv strains (**Supplementary Figures 4A, C**). It also showed high analytical specificity, with no cross-reactivity observed with other mycobacteria or common respiratory pathogens (**Supplementary Figures 4B, D**).

### 3.4 Identification of MDR-TB by the CRISPR-AaCas12b assay

#### 3.4.1 Detection of the *rpoB* 1349C>T mutation

**Figure 2:**
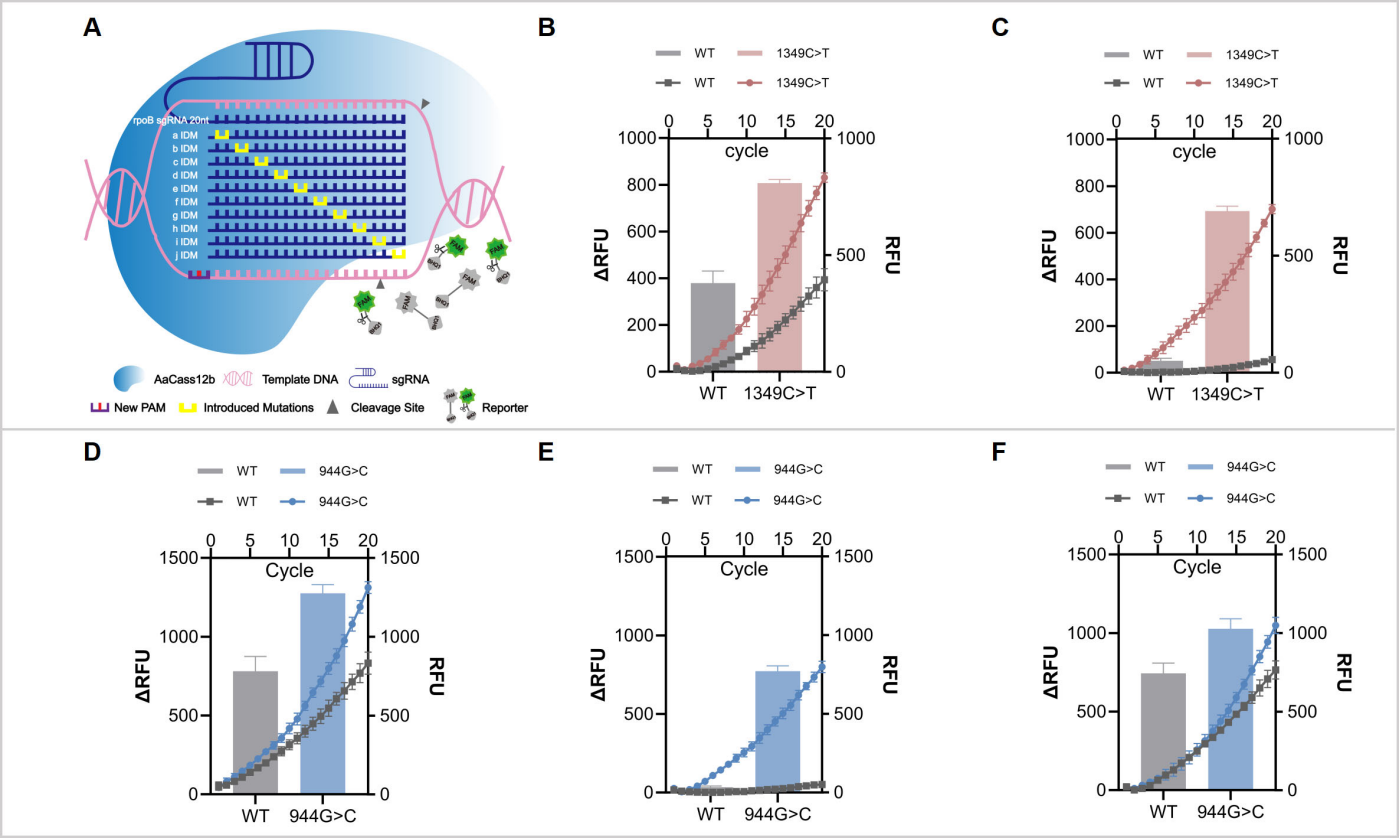
Development of introduction of deliberate mismatches (IDM)-optimized sgRNAs for specific detection of *rpoB* and *katG* mutations using CRISPR-Cas12b. **(A)** Schematic of the IDM strategy for sgRNA optimization. Yellow bars indicate IDM introduced into the sgRNA to suppress off-target cleavage of wildtype (WT) sequences. **(B, C)** Assay performance for the *rpoB* 1349C>T mutation. Panel **(B)** shows detection using the original unmodified 20 nt sgRNA, while panel **(C)** shows detection using the optimized sgRNA with mismatches introduced at spacer positions 13 and 14, which greatly improved specificity with minimal WT signal. **(D, E, F)** Assay performance for the *katG* 944G>C mutation. Panel **(D)** shows detection using the original unmodified 19 nt sgRNA. Panel **(E)** shows the sgRNA with mismatches introduced at spacer positions 7 and 8, which achieved the highest specificity and showed no detectable signal for the WT template. In comparison, panel **(F)** shows the sgRNA with mismatches introduced at spacer positions 9 and 10, which exhibited partial off-target cleavage of the WT sequence. For all panels, the left Y-axis shows the net fluorescence increase (ΔRFU) for the bar plots, and the right Y-axis shows the raw fluorescence intensity (RFU) for the kinetic curves.

Consistent with previous study (Teng et al., 2018; Strecker et al., 2019; Nguyen et al., 2023; Hao et al., 2024), the *rpoB* 1349C>T mutant, which introduces a T residue and generates the novel GTTG PAM, exhibited higher AaCas12b activity with 17–29 nt spacer sequences relative to the wildtype sequence. While, the wildtype PAM, despite containing only a single T nucleotide, still supported detectable AaCas12b cleavage activity with certain spacer lengths. Notably, the 20 nt spacer exhibited the most striking difference in cleavage activity between the mutant and wildtype (**Supplementary Figure 5A**). Then a series of sgRNAs was designed with IDM across its 20 bases proximal to the PAM site in the spacer region (**Figure 2A**). The results showed that the sgRNA with mutations at 13 and 14 nt site following the NTTG PAM showing higher fluorescence signal to *rpo*B 1349C>T comparable with the wildtype (**Figures 2B, C**). The image of AGE results was consistent with the fluorescence signal monitoring results (**Supplementary Figures S5B, C**).

#### 3.4.2 Detection of the *katG* 944G>C mutation

As previously described, a canonical GTTC PAM sequence was identified upstream of the *katG* 944G>C mutation. A panel of 22 nt sgRNAs harboring IDM at various positions throughout the spacer was designed. However, these sgRNAs failed to discriminate between wildtype and mutant alleles, resulting in non-specific cleavage of both templates. (**Supplementary Figure 6A**). To improve specificity, we next tested sgRNAs targeting an alternative ATAC non-canonical PAM, with spacer lengths ranging from 16 to 22 nt. The assay was performed with a shortened reaction time of 5 min to prioritize sgRNAs with rapid, mutation-specific activity. The 19 nt sgRNA targeting the ATAC PAM showed the most promising signal difference between wildtype and mutant (**Supplementary Figure 6B**), though residual activity against the wildtype template was still observed. We therefore further optimized this 19 nt sgRNA by introducing IDM at distinct positions within the spacer (**Supplementary Figure 7A**). This screening identified the sgRNA with mismatches at spacer positions 7 and 8 as the most specific variant, which robustly cleaved the mutant template, but showed no detectable activity against the WT template (**Figures 2D–F**; **Supplementary Figures 7B, C**).

### 3.5 Clinical validation of the CRISPR-AaCas12b TB/MDR-TB assay

**Figure 3:**
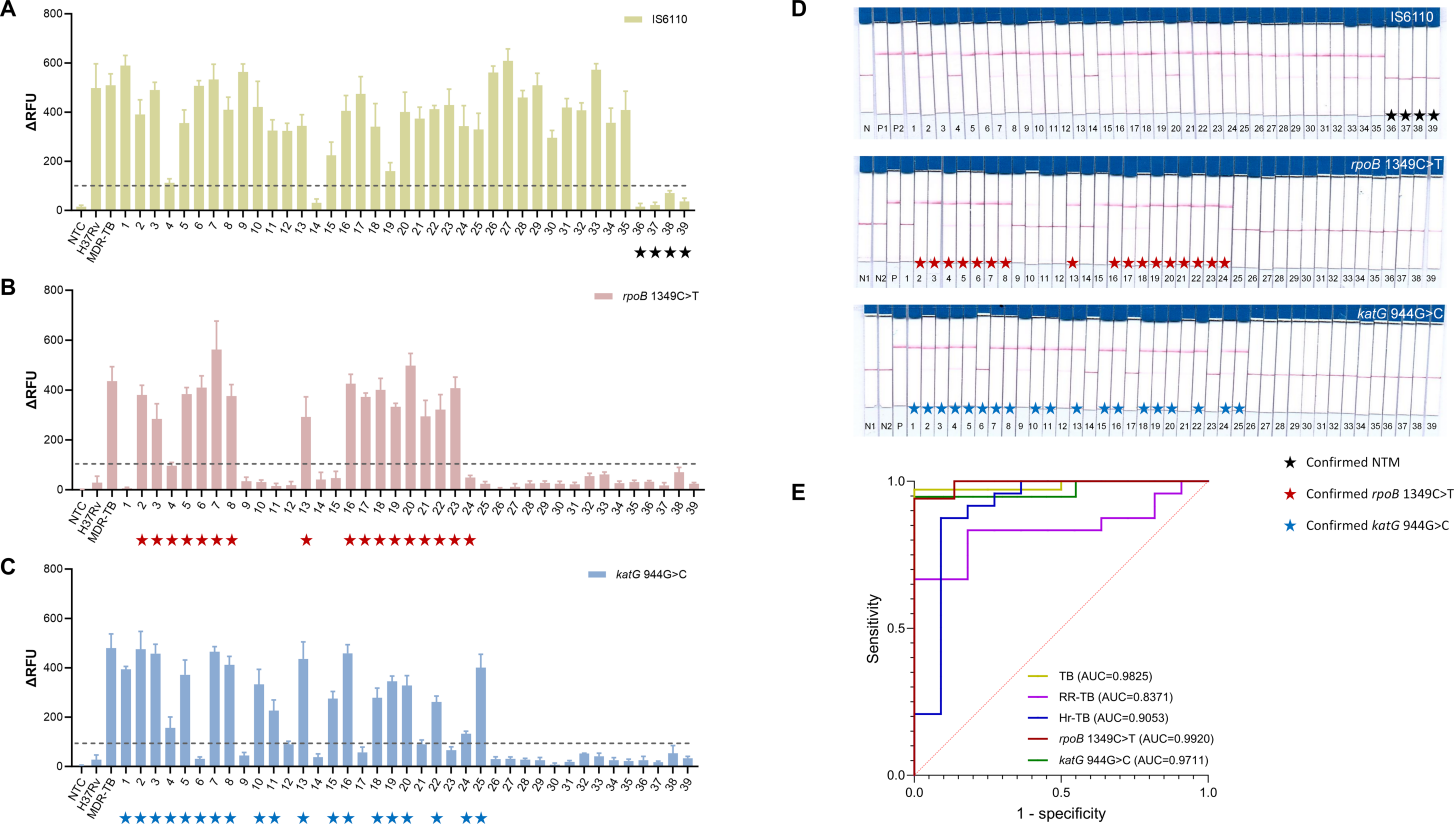
Clinical performance of the multiplex-RPA CRISPR-AaCas12b assay. **(A–C)** Fluorescence detection of clinical samples. **(A)** MTB detection targeting the *IS6110* locus. **(B)** Rifampicin-resistant TB (RR-TB) detection targeting the *rpoB* 1349C>T mutation. **(C)** Isoniazid-resistant TB (Hr-TB) detection targeting the *katG* 944G>C mutation. The dashed line indicates the assay thresholds. Stars mark confirmed samples: black = non-tuberculous mycobacteria (NTM), red = *rpoB* 1349C>T, blue = *katG* 944G>C. **(D)** Lateral flow chromatography (LFC) readout. The upper line corresponds to cleaved reporter (positive signal); the lower line corresponds to uncleaved probe (negative control). Stars mark the same confirmed samples as in **(A–C)**. **(E)** ROC curve analysis. Diagnostic performance for MTB, RR-TB, Hr-TB, *rpoB* 1349C>T, and *katG* 944G>C.

**Figure 4:**
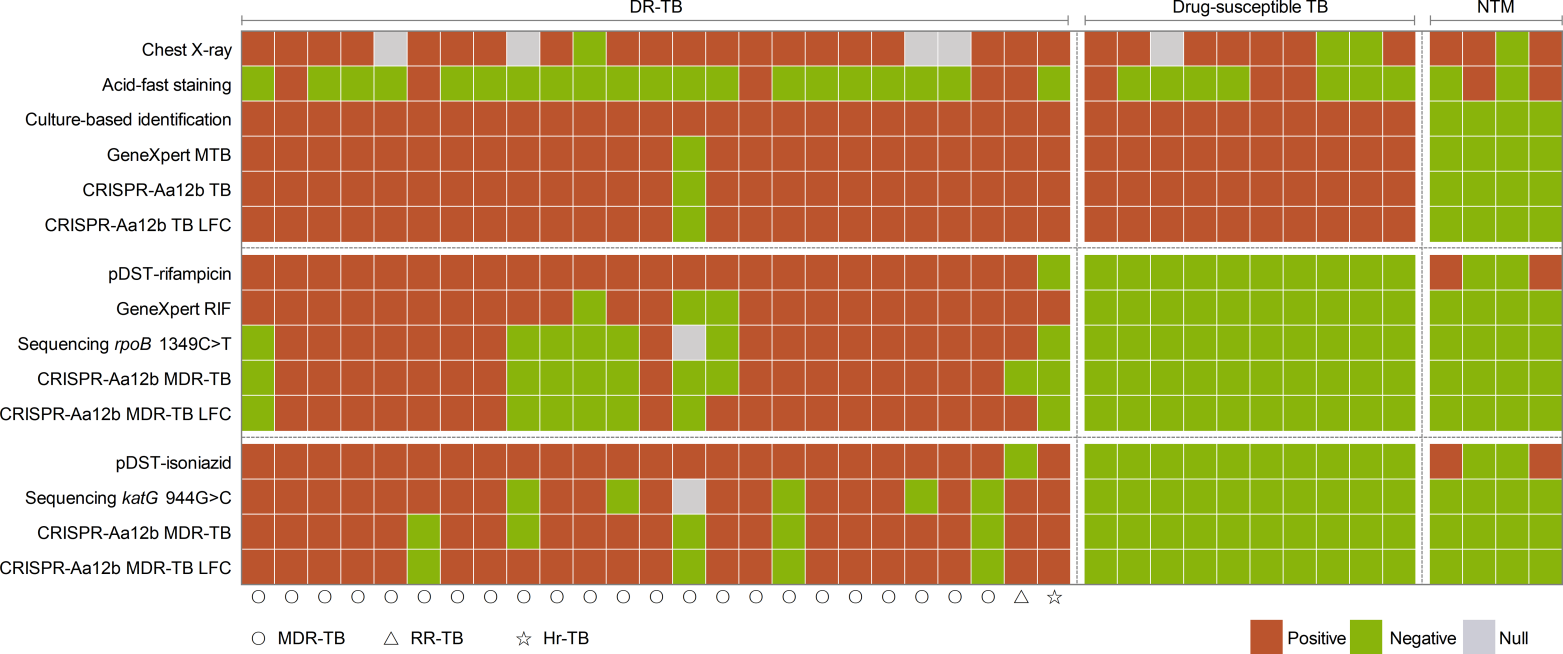
Diagnostic performance of the CRISPR-Aa12b assay in clinical specimens. DR-TB, drug-resistant tuberculosis; NTM, Non-tuberculous Mycobacteria; Strains (left to right): *M. abscessus complex*, *M. kansasii*, *M. gordonae*, *M. malmoense*.

To validate the clinical performance of the established multiplex−RPA CRISPR−AaCas12b assay, we analyzed a total of 48 culture−confirmed clinical specimens individuals with suspected mycobacterial infection. Of these, 39 were culture−positive mycobacteria, including 4 NTM and 35 MTB isolates. The MTB isolates comprised 10 drug-susceptible MTB isolates and 25 DR-TB isolates. (**Supplementary Table 4**). For detection of the *IS6110* sequence, specific to MTB, 34 out of 35 MTB specimens yielded positive signals, while all NTM and all 9 culture-negative specimens tested negative for *IS6110*. This resulted in a sensitivity of 97.1% and a specificity of 100%, which is consistent with the results obtained using the GeneXpert assay (**Figures 3A, E**, **4**; **Supplementary Table 5**).

Of the 48 specimens, 35 were culture-confirmed MTB isolates, among which 24 were identified as RR-TB by phenotypic drug susceptibility testing (pDST). For these 24 RR-TB specimens, the multiplex-RPA CRISPR-AaCas12b assay detected 16 positive cases, yielded a sensitivity of 66.7% (16/24) and a specificity of 100% (11/11). Among all 48 specimens, 17 harbored the *rpoB* 1349C>T mutation confirmed by sequencing, while the remaining 31 specimens did not carry this specific mutation. Using the sequencing results as the reference standard, the assay achieved a sensitivity of 94.1% (16/17) and a specificity of 100% (31/31) for detection of the *rpoB* 1349C>T mutation. (**Figures 3B, E**, **4**; **Table 1**).

**Table 1:** Diagnostic performance of the multiplex-RPA CRISPR-AaCas12b assay.

Target	Assay	SEN (%)	SPE (%)	PPV (%)	NPV (%)	OA (%)	Kappa
*IS6110* (MTB)	Fluorescence	97.1	100	100	92.9	97.9	0.95
	LFC	97.1	100	100	92.9	97.9	0.95
RR-TB	Fluorescence	66.7	100	100	57.9	77.1	0.54
	LFC	79.2	100	100	68.8	85.7	0.70
Hr-TB	Fluorescence	79.2	90.9	95.0	66.7	82.8	0.64
	LFC	83.3	90.9	95.2	71.4	85.7	0.69
*rpoB* 1349C>T	Fluorescence	94.1	100	100	96.9	97.9	0.95
	LFC	100	93.6	89.5	100	95.8	0.90
*katG* 944G>C	Fluorescence	94.7	93.1	90.0	96.4	93.8	0.87
	LFC	94.7	89.7	85.7	96.3	91.7	0.83

SEN, sensitivity; SPE, specificity; PPV, positive predictive value; NPV, negative predictive value; OA, overall agreement; RR-TB, rifampicin-resistant tuberculosis; Hr-TB, isoniazid-resistant tuberculosis; LFC, lateral flow chromatography. For *IS6110* (MTB), the reference standard was mycobacterial culture; For RR-TB and Hr-TB, the reference standard was phenotypic drug susceptibility testing; For *rpoB* 1349C>T and *katG* 944G>C mutations, the reference standard was Sanger sequencing.

Of the 35 culture-confirmed MTB isolates, 24 were identified as Hr-TB by pDST. The assay identified 19 positive cases, achieving a sensitivity of 79.2% (19/24) and a specificity of 90.9% (10/11). Among all 48 specimens, 19 were confirmed to harbor the *katG* 944G>C mutation by sequencing. For detection of this mutation, 18 of the 19 mutant-positive specimens tested positive by the assay, translating to a sensitivity of 94.7% (18/19) and a specificity of 93.1% (27/29) (**Figures 3C, E**, **4**; **Table 1**). Notably, these performance estimates are based on a limited number of clinical specimens; validation in a larger cohort is warranted.

Unexpectedly, the two specimens (NO. 12 and 21) harboring the *katG* S315N (944G>A) mutation yielded positive signals in the assay, which may reflect off-target effects of the designed sgRNA. While this cross-reactivity suggests potential broader reactivity beyond the originally intended target (*katG* 944G>C), it also indicates a limitation in the assay’s specificity, as the current sgRNA design cannot fully distinguish between the 944G>C and 944G>A variants. Further optimization of the guide RNA would be required to improve allele discrimination and avoid unintended cross-reactivity.

It is worth noting that specimen No. 14 tested positive in MTB culture and pDST, but negative by GeneXpert and failed to generate valid results via Sanger sequencing. In line with these nucleic acid test results, the specimen was not detected by our CRISPR-based assay targeting *IS6110*, *rpoB*, or *katG*. This discrepancy may be related to insufficient MTB nucleic acid template or suboptimal nucleic acid extraction efficiency. Further investigation would be needed to confirm the exact cause. For additional specificity evaluation, nine additional culture-confirmed TB-negative specimens were tested for complementary specificity verification, and their results are provided in the **Supplementary Materials** (**Supplementary Figure 8**).

### 3.6 Portable detection via LFC-Integrated CRISPR-AaCas12b assay

To enable on-site detection of TB and DR-TB, the multiplex-RPA CRISPR-AaCas12b system was integrated with LFC (**Figure 3D**). The LFC assay demonstrated 97.1% sensitivity and 100% specificity for TB diagnosis through *IS6110*-targeted detection of MTB (**Supplementary Table 5**). For drug resistance profiling, the assay exhibited a sensitivity of 79.2% (19/24) and a specificity of 100% (11/11) for RR-TB identification via detection of the *rpoB* 1349C>T mutation. For Hr-TB detection targeting the *katG* 944G>C mutation, the assay achieved a sensitivity of 83.3% (20/24) and a specificity of 90.9% (10/11). Importantly, clinical specimens often harbor a broader spectrum of resistance-associated mutations than those covered in this study. When evaluated against this clinical background, the AaCas12b-based MDR-TB assay maintained robust performance for the two target mutations. It showed a sensitivity of 100% (17/17) and a specificity of 93.6% (29/31) for the *rpoB* 1349C>T mutation, and a sensitivity of 94.7% (18/19) and a specificity of 89.7% (26/29) for the *katG* 944G>C mutation (**Figure 4**; **Table 1**; **Supplementary Table 6**). When combined with the CRISPR-AaCas12b assay, this LFC integration further addresses limitations of conventional molecular tests. It retains the CRISPR system’s high specificity for MTB and drug-resistance mutations, while translating fluorescent cleavage signals into visible, qualitative line results, a critical improvement for resource-limited settings where advanced detection instruments are unavailable.

## 4 Discussion

In this study, targeting the MTB-specific *IS6110* element and key drug-resistance mutations in *rpoB* and *katG*, we developed rapid, convenient, accessible, and simultaneous molecular detection methods for MTB and drug resistance by combining RPA with CRISPR-AaCas12b. When evaluated using clinical specimens, the assay showed favorable diagnostic performance, supporting its potential for practical use in clinical MTB and MDR-TB detection.

To contextualize the utility of our developed platform, we compared it with several representative CRISPR−based TB diagnostic approaches (**Supplementary Table 7**). Although previously reported CRISPR assays offer reliable dual- readout detection, they often involve relatively complex preparation procedures and higher material costs (Zhang et al., 2025). Other CRISPR-LFA (Cheng et al., 2023) or photocontrolled CRISPR systems (Hu et al., 2025) provide excellent analytical performance, yet may require multi-step reactions or specialized equipment. Similarly, the ACURAT platform (Zheng et al., 2024) enables sensitive detection of rifampin resistance mutations but is limited to single-drug resistance screening. In comparison, our system features a simplified procedure and a short assay time of only 30–35 min from RPA amplification to CRISPR detection, and employs commercially available LFC strips combined with universal fluorescent reporters to achieve dual-readout detection. Notably, our assay allows simultaneous identification of MTB and multiple drug resistance mutations without complex preparatory or auxiliary manipulations, thereby enhancing its simplicity and reproducibility. This integrated design strikes a pragmatic balance between detection performance and operational accessibility, addressing the unmet clinical need for comprehensive, field-applicable MDR-TB screening in resource-limited settings.

However, several limitations in the present study should be acknowledged. One major limitation is that the number of clinical specimens evaluated was relatively small, which may limit the generalizability of our findings to more diverse patient populations and clinical settings. In addition, our assay only focuses on two representative drug resistance mutations, *rpoB* 1349C>T and *katG* 944G>C, resulting in a relatively narrow mutation detection spectrum compared with comprehensive sequencing-based methods. Furthermore, the current workflow still requires separate manual operation steps and has not been established as a fully closed-tube system, which may increase both operational complexity and potential cross-contamination risks.

To address these limitations, we have outlined the following directions for future work: First, we will expand the clinical sample cohort to further validate the reliability and applicability of this assay in larger patient populations. Second, we will include more clinically prevalent drug resistance mutations to improve the comprehensiveness of detection. We also plan to integrate artificial intelligence-based strategies to rationalize the design of our detection system toward known drug resistance-associated mutations and explore potential novel clinically relevant resistance loci, with the ultimate goal of expanding the detection panel. Third, we will optimize the workflow toward a fully integrated, closed-tube, one-pot format to simplify operation, reduce contamination risks, and facilitate further clinical translation.

Taken together, the CRISPR-AaCas12b dual-readout system established in this study provides a rapid, simple, and practical strategy for the simultaneous detection of MTB and drug resistance mutations. Such characteristics are key prerequisites for translating molecular diagnostics into routine clinical practice in resource-constrained regions. Despite its current limitations, this platform exhibits promising potential for preliminary MDR-TB screening and could serve as a convenient supplementary tool for conventional diagnostic workflows.

## 5 Conclusion

In conclusion, our CRISPR-AaCas12b dual-readout assay integrates the core advantages of multiplex detection, high specificity, and operational convenience, enabling simultaneous detection of MTB and drug resistance via combined fluorescent and LFC readouts. This platform holds promising potential for supporting the WHO’s End TB Strategy by providing a rapid, accurate, and accessible POCT option for DR-TB screening.

## Data Availability

The partial gene sequences generated from the clinical samples in this study have been deposited in the NCBI GenBank database under accession numbers PZ245066–PZ245133. The original contributions presented in the study are included in the article/**Supplementary Material**. Further inquiries can be directed to the corresponding authors.

## References

[B1] Ajbani K. Rodrigues C. Shenai S. Mehta A. (2011). Mutation detection and accurate diagnosis of extensively drug-resistant tuberculosis: report from a tertiary care center in India. J. Clin. Microbiol. 49, 1588–1590. doi: 10.1128/jcm.00113-11. PMID: 21289142 PMC3122820

[B2] Bosch B. DeJesus M. A. Poulton N. C. Zhang W. Engelhart C. A. Zaveri A. . (2021). Genome-wide gene expression tuning reveals diverse vulnerabilities of M. tuberculosis. Cell 184, 4579–4592.e24. doi: 10.1016/j.cell.2021.06.033. PMID: 34297925 PMC8382161

[B3] Broughton J. P. Deng X. Yu G. Fasching C. L. Servellita V. Singh J. . (2020). CRISPR–cas12-based detection of SARS-coV-2. Nat. Biotechnol. 38, 870–874. doi: 10.1038/s41587-020-0513-4. PMID: 32300245 PMC9107629

[B4] Caulfield A. J. Richter E. Brown-Elliott B. A. Wengenack N. L. (2023). “ Mycobacterium: Laboratory Characteristics of Slowly Growing Mycobacteria Other than Mycobacterium tuberculosis,” in ClinMicroNow2023 (Hoboken, NJ: ASM Press/Wiley), 1–18. doi: 10.1002/9781683670438.mcm0032

[B5] Cheng M. Tan C. Xiang B. Lin W. Cheng B. Peng X. . (2023). Chain hybridization‐based CRISPR‐lateral flow assay enables accurate gene visual detection. Anal. Chim. Acta 1270, 341437. doi: 10.1016/j.aca.2023.341437. PMID: 37311609

[B6] Dorman S. E. Schumacher S. G. Alland D. Nabeta P. Armstrong D. T. King B. . (2018). Xpert MTB/RIF Ultra for detection of Mycobacterium tuberculosis and rifampicin resistance: a prospective multicentre diagnostic accuracy study. Lancet Infect. Dis. 18, 76–84. doi: 10.1016/s1473-3099(17)30691-6. PMID: 29198911 PMC6168783

[B7] DRAGdb: Drug Resistance Associated Genes. Available online at: https://bicresources.jcbose.ac.in/ssaha4/drag/index.php (Accessed February 24, 2025).

[B8] Gan T. Yu J. Deng Z. He J. (2024). ERA-CRISPR/Cas12a system: a rapid, highly sensitive and specific assay for Mycobacterium tuberculosis. Front. Cell. Infect. Microbiol. 14. doi: 10.3389/fcimb.2024.1454076. PMID: 39233906 PMC11371737

[B9] Guo Y. Yang J. Wang H. Sha W. Yu F. (2025). Key resistance-associated mutations in multidrug-resistant tuberculosis: a genomic study from Shanghai, China, with a focus on aminoglycosides. BMC Microbiol. 25, 702. doi: 10.1186/s12866-025-04446-x. PMID: 41168684 PMC12574157

[B10] Hao W. Cui W. Liu Z. Suo F. Wu Y. Han L. . (2024). A new-generation base editor with an expanded editing window for microbial cell evolution *in vivo* based on CRISPR–Cas12b engineering. Advanced Sci. (Weinheim Baden-Wurttemberg Germany) 11, e2309767. doi: 10.1002/advs.202309767. PMID: 38602436 PMC11165516

[B11] Hu M. Wang Y. Qi W. Zhang Y. Sun J. Cheng M. . (2025). Photocontrolled programmable enzymatic cascade for robust CRISPR diagnostics. J. Am. Chem. Soc 147, 31004–31015. doi: 10.1021/jacs.5c08704. PMID: 40802893

[B12] Jia N. Wang C. Liu X. Huang X. Xiao F. Fu J. . (2023). A CRISPR-Cas12a-based platform for ultrasensitive rapid highly specific detection of Mycobacterium tuberculosis in clinical application. Front. Cell. Infect. Microbiol. 13. doi: 10.3389/fcimb.2023.1192134. PMID: 37287467 PMC10242030

[B13] Kechin A. Oscorbin I. Cherednichenko A. Khrapov E. Schwartz Y. Stavitskaya N. . (2023). Selection of IS6110 conserved regions for the detection of Mycobacterium tuberculosis using qPCR and LAMP. Arch. Microbiol. 205, 71. doi: 10.1007/s00203-023-03410-5. PMID: 36688992

[B14] Kocagöz T. Yilmaz E. Ozkara S. Kocagöz S. Hayran M. Sachedeva M. . (1993). Detection of Mycobacterium tuberculosis in sputum samples by polymerase chain reaction using a simplified procedure. J. Clin. Microbiol. 31, 1435–1438. doi: 10.1128/jcm.31.6.1435-1438.1993 8314982 PMC265557

[B15] Lessells R. J. Cooke G. S. McGrath N. Nicol M. P. Newell M. L. Godfrey-Faussett P. (2017). Impact of point-of-care Xpert MTB/RIF on tuberculosis treatment initiation. A cluster-randomized trial. Am. J. Respir. Crit. Care Med. 196, 901–910. doi: 10.1164/rccm.201702-0278oc. PMID: 28727491 PMC5649979

[B16] Li L. Li S. Wu N. Wu J. Wang G. Zhao G. . (2019). HOLMESv2: A CRISPR-Cas12b-assisted platform for nucleic acid detection and DNA methylation quantitation. ACS Synth. Biol. 8, 2228–2237. doi: 10.1021/acssynbio.9b00209 31532637

[B17] Li Q. Wang N. Pang M. Miao H. Dai X. Li B. . (2024). Rapid and highly sensitive detection of Mycobacterium tuberculosis utilizing the recombinase aided amplification-based CRISPR-Cas13a system. Microorganisms 12, 1507. doi: 10.3390/microorganisms12081507. PMID: 39203350 PMC11356214

[B18] Myhrvold C. Freije C. A. Gootenberg J. S. Abudayyeh O. O. Metsky H. C. Durbin A. F. . (2018). Field-deployable viral diagnostics using CRISPR-Cas13. Science 360, 444–448. doi: 10.1126/science.aas8836. PMID: 29700266 PMC6197056

[B19] Naidoo K. Dookie N. (2022). Can the GeneXpert MTB/XDR deliver on the promise of expanded, near-patient tuberculosis drug-susceptibility testing? Lancet Infect. Dis. 22, e121–e127. doi: 10.1016/s1473-3099(21)00613-7. PMID: 35227392

[B20] Nguyen L. T. Macaluso N. C. Pizzano B. L. M. Cash M. N. Spacek J. Karasek J. . (2022). A thermostable Cas12b from Brevibacillus leverages one-pot discrimination of SARS-CoV-2 variants of concern. eBioMedicine 77, 103926. doi: 10.1016/j.ebiom.2022.103926. PMID: 35290826 PMC8917962

[B21] Nguyen L. T. Rananaware S. R. Yang L. G. Macaluso N. C. Ocana-Ortiz J. E. Meister K. S. . (2023). Engineering highly thermostable Cas12b via de novo structural analyses for one-pot detection of nucleic acids. Cell Rep. Med. 4, 101037. doi: 10.1016/j.xcrm.2023.101037. PMID: 37160120 PMC10213852

[B22] Organization GWH Global tuberculosis report 2025–2025. Available online at: https://www.who.int/teams/global-programme-on-tuberculosis-and-lung-health/tb-reports/global-tuberculosis-report-2025 (Accessed January 17, 2026).

[B23] Pardee K. Green A. A. Takahashi M. K. Braff D. Lambert G. Lee J. W. . (2016). Rapid, low-cost detection of Zika virus using programmable biomolecular components. Cell 165, 1255–1266. doi: 10.1016/j.cell.2016.04.059. PMID: 27160350

[B24] Patchsung M. Jantarug K. Pattama A. Aphicho K. Suraritdechachai S. Meesawat P. . (2020). Clinical validation of a Cas13-based assay for the detection of SARS-CoV-2 RNA. Nat. Biomed. Eng. 4, 1140–1149. doi: 10.1038/s41551-020-00603-x. PMID: 32848209

[B25] Peng L. Fang T. Cai Q. Li H. Li H. Sun H. . (2023). Rapid detection of Mycobacterium tuberculosis in sputum using CRISPR-Cas12b combined with cross-priming amplification in a single reaction. J. Clin. Microbiol. 62, e00923-23. doi: 10.1128/jcm.00923-23. PMID: 38112450 PMC10793277

[B26] Quan Z. Qiu Y. Li M. Tian F. Qu R. Tang Y.-W. . (2024). Pooling sputum samples for the Xpert MTB/RIF Ultra assay: a sensitive and effective screening strategy. Tuberculosis 149, 102575. doi: 10.1016/j.tube.2024.102575. PMID: 39541856

[B27] Rostamian M. Kooti S. Abiri R. Khazayel S. Kadivarian S. Borji S. . (2023). Prevalence of Mycobacterium tuberculosis mutations associated with isoniazid and rifampicin resistance: a systematic review and meta-analysis. J. Clin. Tuberculosis Other Mycobacterial Dis. 32, 100379. doi: 10.1016/j.jctube.2023.100379. PMID: 37389010 PMC10302537

[B28] Strecker J. Jones S. Koopal B. Schmid-Burgk J. Zetsche B. Gao L. . (2019). Engineering of CRISPR-Cas12b for human genome editing. Nat. Commun. 10, 212. doi: 10.1038/s41467-018-08224-4. PMID: 30670702 PMC6342934

[B29] Teng F. Cui T. Feng G. Guo L. Xu K. Gao Q. . (2018). Repurposing CRISPR-Cas12b for mammalian genome engineering. Cell Discov. 4, 63. doi: 10.1038/s41421-018-0069-3. PMID: 30510770 PMC6255809

[B30] Teng F. Cui T. Gao Q. Guo L. Zhou Q. Li W. (2019a). Artificial sgRNAs engineered for genome editing with new Cas12b orthologs. Cell Discov. 5, 23. doi: 10.1038/s41421-019-0091-0. PMID: 31016029 PMC6476878

[B31] Teng F. Guo L. Cui T. Wang X. G. Xu K. Gao Q. . (2019b). CDetection: CRISPR-Cas12b-based DNA detection with sub-attomolar sensitivity and single-base specificity. Genome Biol. 20, 132. doi: 10.1186/s13059-019-1742-z. PMID: 31262344 PMC6604390

[B32] The Comprehensive Antibiotic Resistance Database. Available online at: https://card.mcmaster.ca/ (Accessed February 27, 2025).

[B33] Wang W. Du H. Dai C. Ma H. Luo S. Wang X. . (2025). Amplification-free detection of Mycobacterium tuberculosis using CRISPR-Cas12a and graphene field-effect transistors. Nanoscale 17, 4603–4609. doi: 10.1039/d4nr03852e. PMID: 39810563

[B34] Zhang L. Bai H. Zhang C. He X. Zou J. Bai W. . (2025). Dual-mode sensing of Mycobacterium tuberculosis with DNA-functionalized gold nanoparticles and asymmetric RPA-triggered PAM-free CRISPR system. Sensors Actuators B. Chem. 424, 136920. doi: 10.1016/j.snb.2024.136920. PMID: 38826717

[B35] Zheng R. Zhang L. Yu C. Parvin R. Yang S. Yao D. . (2024). ACURAT: Advancing tuberculosis detection through assembled PCR & CRISPR for ultra-sensitive Rifampin-resistant analysis testing. Chem. Eng. J. 494, 152712. doi: 10.1016/j.cej.2024.152712. PMID: 38826717

