# Supplementary material for: Simultaneous molecular detection of Mycobacterium tuberculosis and multidrug resistance using CRISPR-AaCas12b-based nucleic acid assay
Source: Front Cell Infect Microbiol. 2026 May 19;16:1844184. doi: 10.3389/fcimb.2026.1844184 (PMC13226185; doi:10.3389/fcimb.2026.1844184)
Supplement: Supplementary file 1 [file DataSheet1.pdf]

Supplementary Material

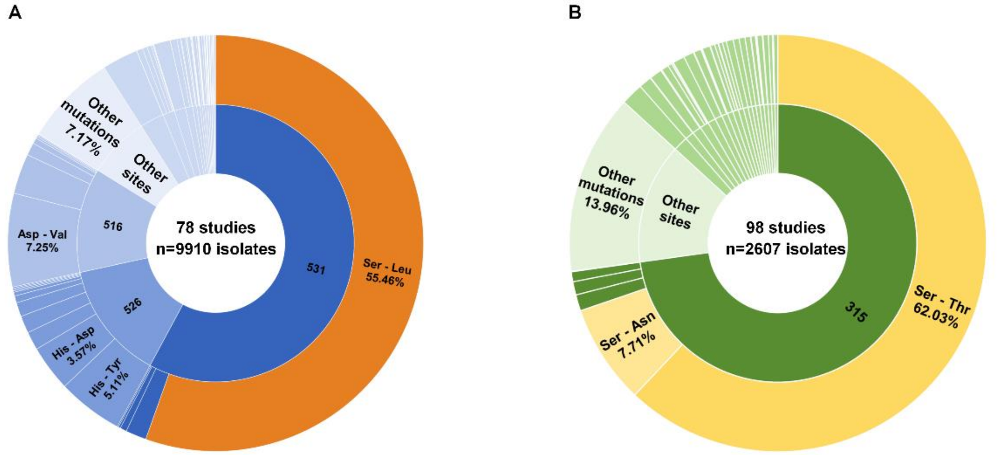

Supplementary Figure 1. Overview of MTB drug-resistance mutation profiles. A) Sunburst plot showing the distribution of *rpoB* mutations associated with rifampicin resistance. **B)** Sunburst plot showing the distribution of *katG* mutations associated with isoniazid resistance. Only mutations with a frequency > 3% are labeled individually. All other variants with a frequency ≤ 3% are depicted as unlabeled and fine-grained segments in the plot, while the "Other mutations" and "Other sites" categories represent the collective summary of the low-frequency variants that were not enumerated individually.

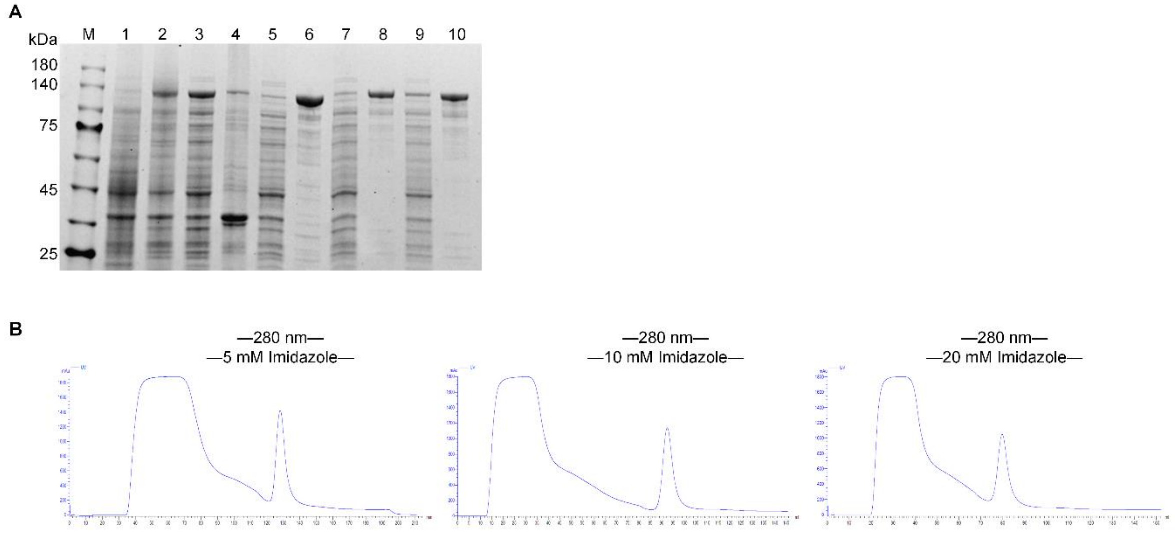

Supplementary Figure 2. Expression and purification of CRISPR-AaCas12b. A) Coomassie Brilliant Blue staining of AaCas12b protein expression and purification. Lane M, Protein marker; Lane 1, Negative control (uninduced bacterial suspension); Lane 2, Bacterial suspension induced by IPTG; Lane 3, Supernatant after ultrasonic disruption and centrifugation; Lane 4, Precipitate after ultrasonic disruption and centrifugation; Lanes 5, 7, 9: Flow-through fractions eluted with 5 mM, 10 mM, and 20 mM imidazole, respectively; Lanes 6, 8, 10: Eluted fractions obtained with 5 mM, 10

mM, and 20 mM imidazole, respectively. **B)** Absorbance profiles (280 nm) of AaCas12b protein eluted with different imidazole concentrations.

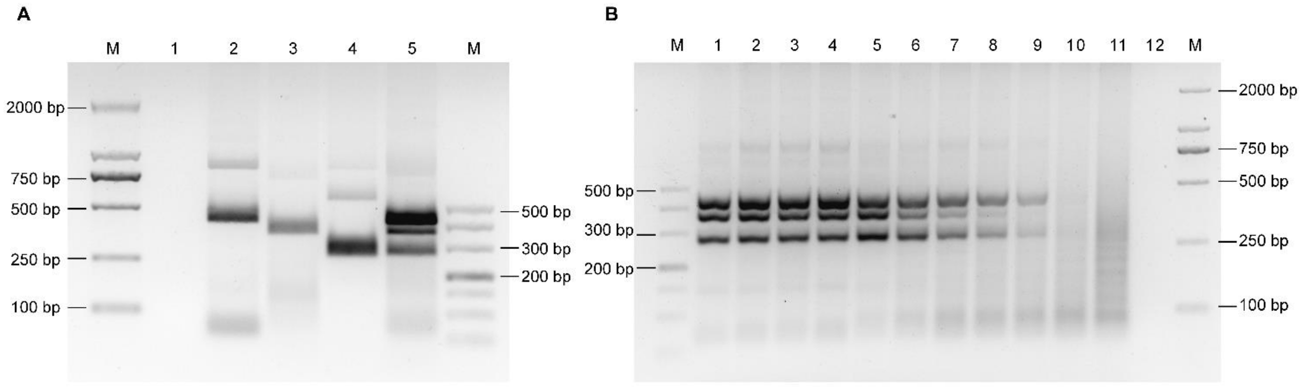

Supplementary Figure 3. RPA results of *M. tuberculosis* H37Rv. A) Agarose gel electrophoresis (AGE) of RPA products. Left lane M, DL2000 DNA marker; Lane 1, Negative control (no primers added); Lane 2, RPA product targeting *IS6110*; Lane 3, RPA product targeting *rpoB*; Lane 4, RPA product targeting *katG*; Lane 5, Multiplex-RPA product targeting *IS6110*, *rpo*B and *kat*G. Right lane M, DL500 DNA marker. **B)** AGE of optimized RPA products from serially tenfold-diluted *M. tuberculosis* H37Rv templates. Left lane M: DL500 DNA marker; Lane 1: Template at 0.5 McFarland turbidity standard; Lanes 2–10: Serially tenfold-diluted templates; Lane 11: No-template control (NTC); Lane 12: Negative control (no RPA primers added); Right lane M: DL2000 DNA marker.

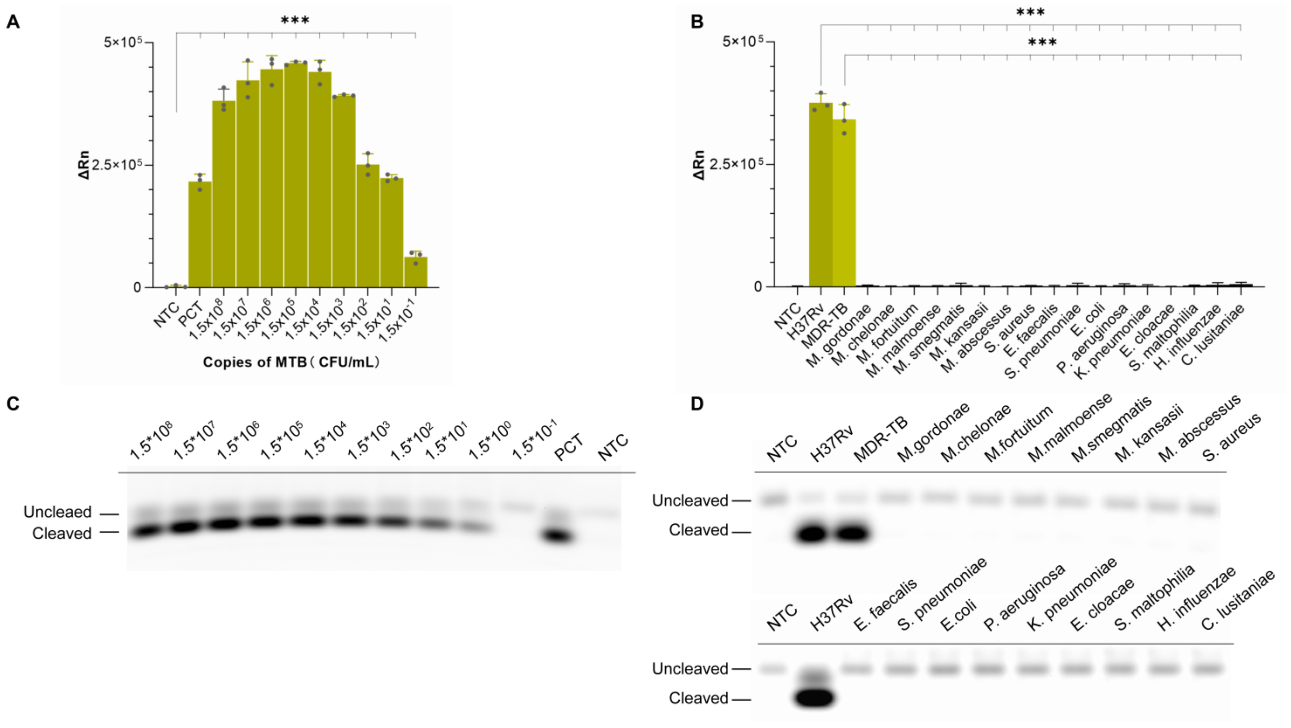

Supplementary Figure 4. CRISPR-AaCas12b assay for MTB detection. A) Fluorescence-based sensitivity of the CRISPR-AaCas12b MTB assay, ****P* < 0.001. **B)** Fluorescence-based specificity of the CRISPR-AaCas12b MTB, ****P* < 0.001. **C)** Urea-polyacrylamide gel electrophoresis (Urea-

PAGE) analysis of the assay’s sensitivity; Uncleaved/Cleaved = ssDNA reporters cleavage status. **D)** Urea-PAGE analysis of the assay’s specificity.

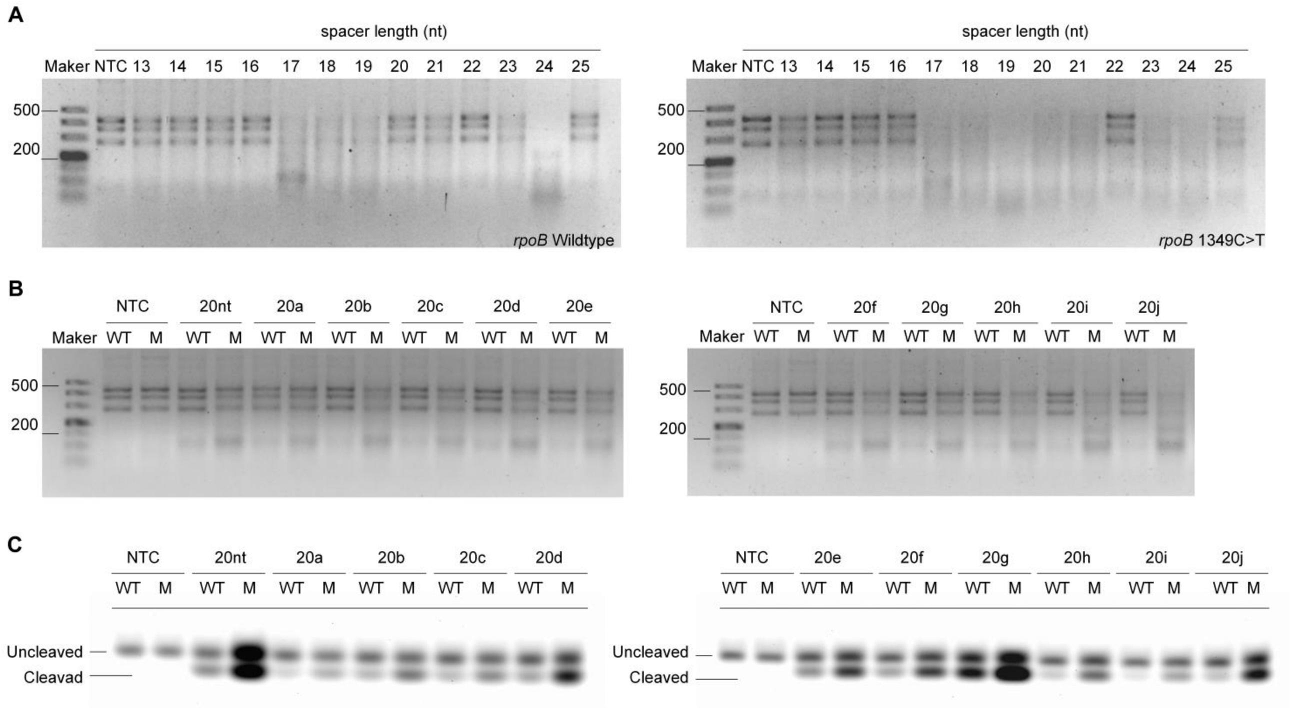

Supplementary Figure 5. Detection of *rpoB* 1349C>T via CRISPR-AaCas12b MDR-TB assay. A) AGE analysis of discrimination of *rpoB* wildtype (left panel) and 1349C>T (right panel) mutant alleles using sgRNAs with 13-25 nt spacer length. **B)** AGE analysis of position-dependent introduction of deliberate mismatches (IDM) effects on 20-nt spacer sgRNAs tested for *rpoB* wildtype (WT) and 1349C>T (M) mutant. **C)** Urea-PAGE analysis of position-dependent IDM effects of 20-nt spacer sgRNAs tested for *rpoB* wildtype (WT) and 1349C>T (M); Uncleaved/Cleaved = ssDNA reporters cleavage status.

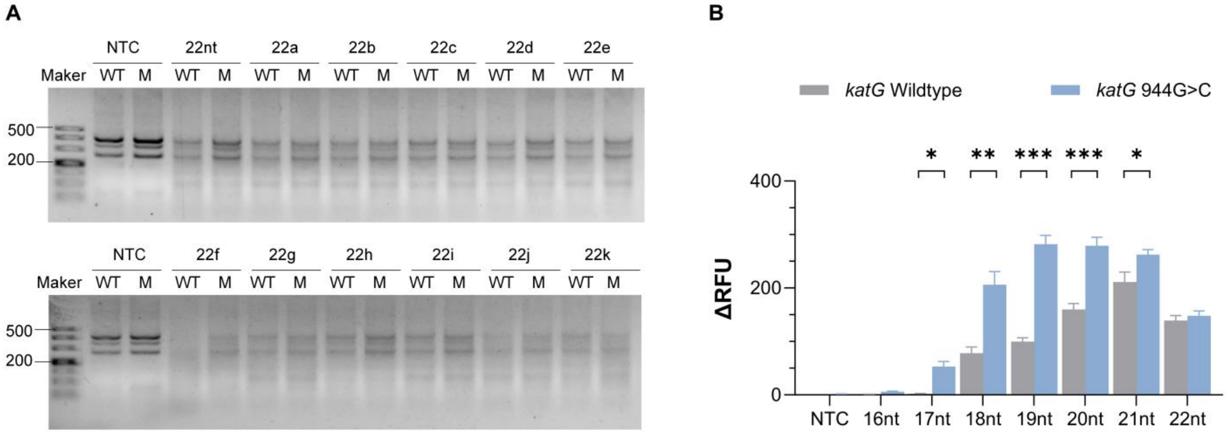

Supplementary Figure 6. Detection of *katG* 944G>C via CRISPR-AaCas12b MDR-TB assay. A) AGE analysis of position-dependent IDM effects on 22-nt spacer sgRNAs (NTTN PAM-adjacent) tested for *katG* wildtype (WT) and 944G>C (M). **B)** Fluorescence signal analysis of *katG* wildtype

and 944G>C detection using sgRNAs (non-canonical PAM-adjacent) with 16–22 nt spacer length; ΔRFU = net fluorescence increase; *P < 0.05, **P < 0.01, ***P < 0.001.

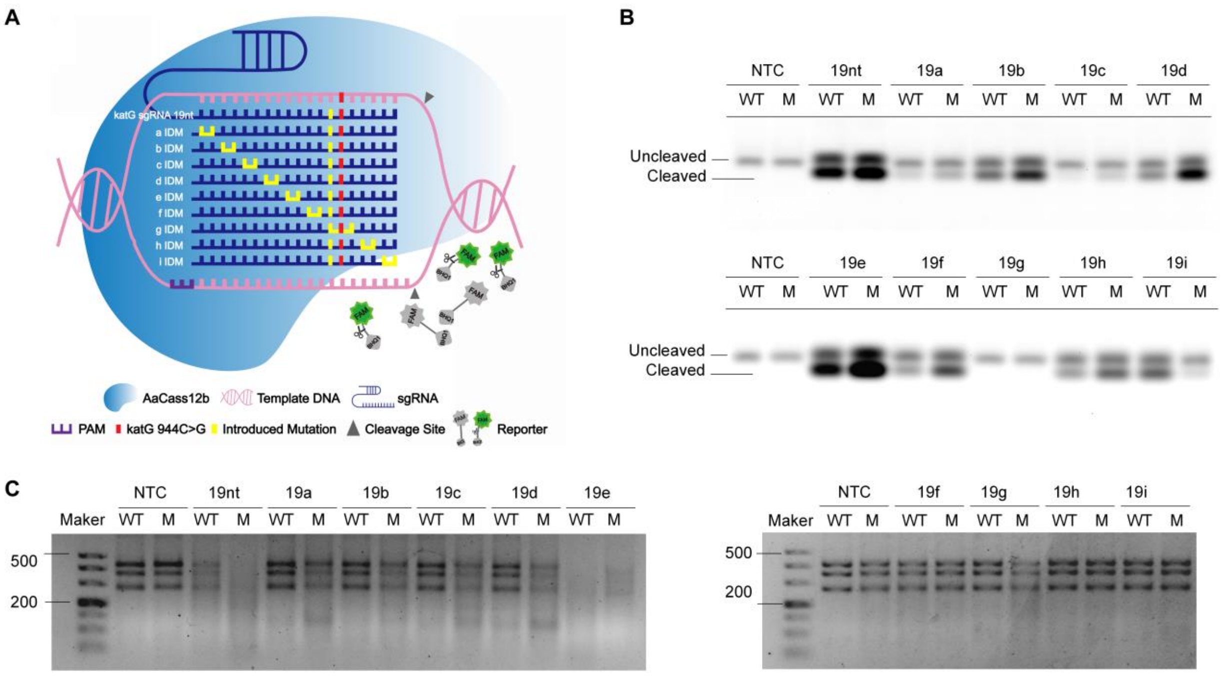

Supplementary Figure 7. Detection of *katG* 944G>C mutation via CRISPR-AaCas12b-based MDR-TB assay (ATAC PAM). A) Schematic diagram of position-dependent IDM for sgRNA design targeting the *katG* 944G>C locus. **B)** Urea-PAGE analyzed of position-dependent IDM effects of 19-nt spacer sgRNAs tested for *katG* wildtype (WT) and 944G>C (M). **C)** AGE analyzed of position-dependent IDM effects of 19-nt spacer sgRNAs tested for *katG* wildtype (WT) and 944G>C (M).

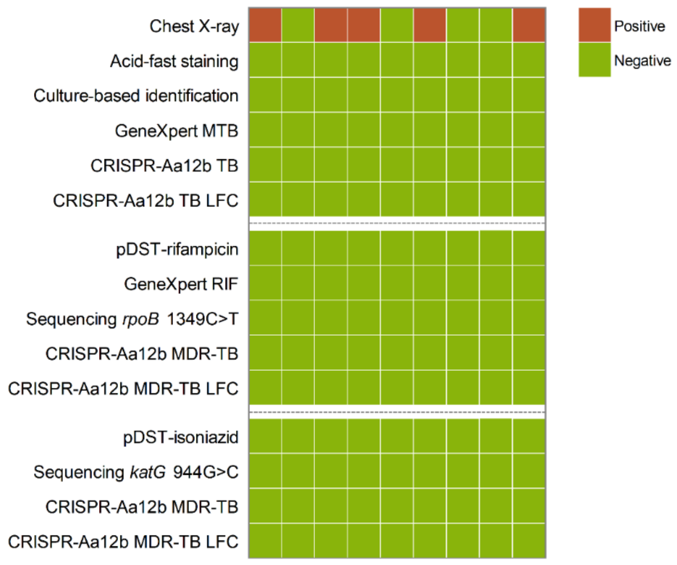

Supplementary Figure 8. Diagnostic performance of the CRISPR-AaCas12b assay in 9 additional clinical specimens. This heatmap summarizes results for 9 non-mycobacterial clinical

specimens. Rows show diagnostic methods targeting TB, rifampicin resistance (*rpoB* 1349C>T), and isoniazid resistance (*katG* 944G>C). Red indicates a positive result, and green indicates a negative result.
